# A Decision Tree Based Methodology for Evaluating Creativity in Engineering Design

**DOI:** 10.3389/fpsyg.2019.00032

**Published:** 2019-01-25

**Authors:** Trina C. Kershaw, Sankha Bhowmick, Carolyn Conner Seepersad, Katja Hölttä-Otto

**Affiliations:** ^1^Department of Psychology, University of Massachusetts Dartmouth, Dartmouth, MA, United States; ^2^Department of Mechanical Engineering, University of Massachusetts Dartmouth, Dartmouth, MA, United States; ^3^Department of Mechanical Engineering, The University of Texas at Austin, Austin, TX, United States; ^4^Design Factory, Department of Mechanical Engineering, Aalto University, Helsinki, Finland

**Keywords:** creativity, engineering design, decision tree, creative products, creativity measurement, creativity metrics

## Abstract

Multiple metrics have been proposed to measure the creativity of products, yet there is still a need for effective, reliable methods to assess the originality of new product designs. In the present article we introduce a method to assess the originality of concepts that are produced during idea generation activities within engineering design. This originality scoring method uses a decision tree that is centered around distinguishing design innovations at the system level. We describe the history and the development of our originality scoring method, and provide evidence of its reliability and validity. A full protocol is provided, including training procedures for coders and multiple examples of coded concepts that received different originality scores. We summarize data from over 500 concepts for garbage collection systems that were scored by Kershaw et al. (2015). We then show how the originality scoring method can be applied to a different design problem. Our originality scoring method, the Decision Tree for Originality Assessment in Design (DTOAD), has been a useful tool to identify differences in originality between various cohorts of Mechanical Engineering students. The DTOAD reveals cross-sectional differences in creativity between beginning and advanced students, and shows longitudinal growth in creativity from the beginning to the end of the undergraduate career, thus showing how creativity can be influenced by the curriculum. The DTOAD can be applied to concepts produced using different ideation procedures, including concepts produced both with and without a baseline example product, and concepts produced when individuals are primed to think of different users for their designs. Finally, we show how our the DTOAD compares to other measurements of creativity, such as novelty, fixation, and remoteness of association.

## Introduction

There are many ways to define creativity (Batey, 2012), but a common definition is that creativity involves the production of ideas that are novel and useful (Sternberg and Lubart, 1999). There are also many ways to narrow the focus of creativity research, but a common framework involves the 4 Ps: person, process, press (environment), and product (Runco, 2004; Cropley et al., 2017; but see Kozbelt et al., 2010 for 6 Ps, which add persuasion and potential, or Lubart, 2017, who proposes the 7 Cs of creators, creating, collaborations, contexts, creations, consumption, and curricula). Our focus is on the evaluation of the creative product; that is, the outcome of the creative process. According to Plucker and Makel (2010), evaluation of creative products is the “gold standard” of creativity assessment. Evaluation of creative products can take different forms depending on the nature of the product and the way in which creativity is defined. For example, objective scoring is common for divergent thinking test responses, while a panel of expert judges is frequently used to subjectively score artistic (c.f. Getzels and Csikzentmihalyi, 1976) or musical (Beaty et al., 2013) works.

### Evaluating Creative Products in Psychology

While objective evaluations of creative products have been utilized in psychology, such as a citation index of composers’ works (Hass and Weisberg, 2015), subjective evaluations are far more prevalent. A common method of evaluating creative products within the psychology literature is the consensual assessment technique (CAT; Amabile, 1982). The CAT involves subjective ratings of creative products from a particular domain by a group of people who are knowledgeable within that domain. Amabile (1982) provides specific guidelines for products to be evaluated using the CAT, such as choosing target tasks that are open-ended, allow novel responses, and result in a product that can be judged. Amabile calls for a set of judges with experience in the target domain who rate the resulting products independently, in a random order, and versus each other rather than versus a standard. She also recommends that products are rated on multiple dimensions, such as technical aspects and aesthetic appeal, rather than purely on creativity.

The CAT is a popular method for rating products that have been produced using what has often been called *little-c creativity* (cf. Kozbelt et al., 2010), such as drawings produced by children (Rostan, 2010; Storme et al., 2014) or college students (Dollinger and Shafran, 2005), collages made by children or college students (Amabile, 1982), short stories written by college students (Kaufman et al., 2013), and improvised jazz performances (Beaty et al., 2013). As noted by Baer and McKool (2009), one of the advantages of the CAT is that it can be used in multiple settings because it is not tied to a particular theory. Further, other advantages are that the CAT shows high inter-rater reliability using multiple statistics, including Chronbach’s alpha, Spearman-Brown correlations, or intraclass correlations, and that the CAT does not display any differences in ratings obtained related to race, ethnicity, or gender (Baer and McKool, 2009).

Although the CAT has wide application in psychology, there are downsides to its administration. First, the high inter-rater reliabilities that are reported are in part due to using a large number of judges. For example, Amabile (1982) reports using 6–15 judges per study. Although good reliability has been found with as few as three judges (e.g., Rostan, 2010; Beaty et al., 2013), groups of 15 judges (c.f. e.g., Kaufman et al., 2013; Jeffries, 2017) are not uncommon. Inter-rater reliability statistics are influenced by the number of raters (Gwet, 2014), so it is possible that the agreement levels reported in published research may be inflated.

Second, the CAT requires that selected judges should have experience in the domain that they are judging (Amabile, 1982). As noted by Baer and McKool (2009), this is usually interpreted as a need to have expert judges who can rely on their knowledge of the domain. Finding and compensating appropriate experts is a further strain on researchers. A recent paper questioned the need for expert judges: Kaufman et al. (2013) found that expert judges (professional writers) provided ratings of short stories that were highly correlated with quasi-expert judges (creativity researchers, advanced elementary education or English majors, and English teachers) and moderately correlated with novice judges (college students). This finding may be dependent on domain, however: in a second study, Kaufman et al. (2013) showed that quasi-expert judges (first-year engineering students) and novice judges (students in an introductory psychology courses) did not provide creativity ratings of mousetrap designs that were sufficiently correlated with expert judges’ (professional engineers) ratings.

It is possible that greater agreement could be achieved if judges received training, but that would go against another requirement of the CAT. Amabile (1982) specifies that judges should not be trained by researchers to agree with each other, and that they should not receive any definition of creativity. Judges’ knowledge of the respective domain should provide enough information for them to know what is creative. This tenet of the CAT has been challenged by two recent papers. Dollinger and Shafran (2005) found that providing non-expert judges (psychology research assistants) with a 4-min review of previously rated drawings boosted their inter-rater agreement with professional artists on ratings of details and overall creativity of drawings, compared to a previous study contrasting untrained non-experts to experts. Storme et al. (2014) contrasted trained novice judges and control novice judges (all students in an introductory psychology course) with expert judges (elementary school art teachers). The trained group was provided with specific definitions of creativity, rated a practice set of drawings, and compared their ratings on the practice set to experts’ ratings. On a new set of drawings, the trained group showed a higher level of agreement with the expert judges than the control group.

Overall, while the CAT (Amabile, 1982) has wide use in psychology, and is a successful way to evaluate creative products (cf. Baer and McKool, 2009), there are downsides to its administration, such as the number of judges required, the expertise of the judges, and a requirement that judges should not be trained. While various researchers have developed alternative ways of using the CAT (cf. Dollinger and Shafran, 2005; Kaufman et al., 2013; Storme et al., 2014), there are applications where it has been less useful. For example, Jeffries (2017) reports varying levels of inter-rater reliability depending on the target graphic design task that is used with the CAT. While simpler tasks, such as manipulating text to creatively express one word, had high levels of inter-rater reliability, more complex tasks, such as designing a t-shirt graphic, had unacceptable levels of inter-rater reliability. Further, and more germane to our research, Kaufman et al. (2013, Study 2) questioned the use of the CAT for evaluating what Cropley and Cropley (2010) refer to as “functional creativity” – the generation of concrete, useful products. Cropley (2015) even goes so far as to suggest that the CAT may be better used to measure creativity of people rather than products. There are, however, alternative methods for evaluating creative products within the engineering literature.

### Evaluating Creative Products in Engineering

While creative products generated in engineering settings should meet the common creativity criteria of being novel and useful (Sternberg and Lubart, 1999), it is possible that engineers are generating new ideas in different ways than are typically measured within psychology studies. Cropley et al. (2017) suggest that engineering creativity involves first determining a function and then finding ways, referred to as forms, that this function could be satisfied. While all creative products research goes beyond typical divergent thinking tests, research using creative products in engineering tends to employ different kinds of samples and different modes of evaluation. While some creative product studies in psychology involve the evaluation of products generated by individuals with high domain knowledge, such as advanced students within a field or domain experts (cf. Getzels and Csikzentmihalyi, 1976; Dunbar, 1997; Beaty et al., 2013), many involve products generated by individuals with low domain knowledge, such as children or undergraduates drawn from a research pool (cf. Amabile, 1982; Dollinger and Shafran, 2005; Kaufman et al., 2013, Study 1; Rostan, 2010; Storme et al., 2014). In contrast, research in engineering creativity tends to involve the evaluation of products generated by individuals with high domain knowledge, such as engineering students at various levels (e.g., Charyton et al., 2008; Chan et al., 2011; Youmans, 2011; Oman et al., 2013; Toh and Miller, 2014) or professional engineers (e.g., Jansson and Smith, 1991, Experiment 4; Moreno et al., 2014; Yilmaz et al., 2014).

While much assessment of creative products within psychology has used the CAT, one common form of assessment of creative products within engineering uses several metrics developed by Shah et al. (2003). Shah et al. (2003) propose metrics for the evaluation of novelty (uniqueness of a single idea generated by one person among a given set of ideas generated by many people), variety (number of different ideas generated by one person), quality (feasibility of meeting design specifications by one or more ideas generated by one person), and quantity (all the ideas generated by one person). The novelty metric is similar to the CAT in that it can be used to evaluate the overall creativity of a product. This metric, however, is applied in a very different way than the CAT. The CAT requires the subjective judgment of creativity by a panel of raters, while the novelty metric is a mostly objective determination of the uniqueness of a product within a particular set of products.

The novelty metric is applied by first decomposing a given product into features based on different functions (Shah et al., 2003). For example, if the creative product were an alarm clock, then the features may include the mode of alarm, the display type, the information shown on the clock, and its energy source (Srivathsavai et al., 2010). Second, product ideas are then described by labeling the expression of each feature. For example, an alarm clock could play a set of songs selected by the user as an alarm, incorporate an LED display, show the time, date, and weather forecast on the clock, and power itself by battery. Third, all described features of a given creative product are compared to the range of features expressed within a set of products. For example, the novelty of a product’s mode of alarm is determined by comparing it to the mode of alarm of all other products within the set. If the mode of alarm is highly unique within the set (e.g., waking a user with a mist of water on the face) it receives a higher novelty score than a mode of alarm that is common with the set (e.g., waking a user with music or a beep). Shah et al.’s (2003) novelty metric can express the uniqueness of a particular feature within a set of creative products, or can combine the uniqueness of the features of a creative product to provide an overall measure of a creative product’s novelty.

Shah et al.’s (2003) metrics are very popular. A recent search on Google Scholar shows over 750 citations of Shah et al.’s (2003) paper. Despite their frequent use, some limitations to Shah et al.’s (2003) metrics have been expressed. For example, Sarkar and Chakrabarti (2011) critique Shah et al.’s (2003) reliance on uniqueness to measure novelty. Srivathsavai et al. (2010) raise the same criticism, noting that creative products within a particular set are not compared to other sets, and are not compared to current products in the market. As noted by Silvia et al. (2008), this is an issue with all rarity scoring methods: creativity is dependent on sample size (the chance of a rare idea with a smaller sample size is higher). Srivathsavai et al. (2010) also found low correlations between raters, average *r* = 0.24, using Shah et al.’s (2003) novelty metric, which contrasts to Shah et al.’s (2003) reported average *r* = 0.62.

An alternative to Shah et al.’s (2003) novelty metric is Charyton et al.’s (2008) Creative Engineering Design Assessment (CEDA; also see Charyton, 2014). The CEDA is a measurement of creative product design in which participants are asked to create designs that incorporate provided three-dimensional objects, satisfy particular functions (ex. designs that produce sound), list potential users for the resulting creative product(s), and generate alternative uses for their creative product(s). Similar to the CAT, judges rate each participant’s resulting creative products for their fluency, flexibility, and originality. For originality, judges are asked to view the product and generate a label that best describes the level of originality, then match that label to the descriptions provided in the CEDA originality metric, which is an 11-point scale that ranges from 0 (dull) to 10 (genius).

Charyton et al. (2008) report high inter-rater reliability, with *r* = 0.84 between two raters, one with a psychology background and one with an engineering background, on the originality scale. In later work, Charyton (2014) reported *r* = 0.59 on the originality scale between five raters, four with an engineering background and one with a psychology background. Neither paper reports the number of creative products that were evaluated to achieve these levels of inter-rater reliability, which calls into question the quality of the scale. In addition, other researchers have had trouble applying the CEDA originality metric to other design problems. As noted by Brown (2014), it may be difficult for judges to determine which label to choose from the metric, as the labels are open to subjective interpretation. For example, Srivathsavai et al. (2010) found low inter-rater agreement between judges, with an average of *r* = 0.35 for the 11-point scale. They also created modified 3- and 4-point originality scales that kept some of the same labels used in the CEDA rubric. These modified scales did not show improved inter-rater correlations, *r* = 0.21 and *r* = 0.29, respectively, but did show an increase in simple agreement over the 11-point scale (3-point scale = 0.68, 4-point scale = 0.57, 11-point scale = 0.20; it should be noted that there was not a statistically significant difference in simple agreement between the 3- and 4-point scales). Srivathsavai et al.’s (2010) results showing better simple agreement with a smaller set of alternatives is similar to findings showing that higher inter-rater agreement is reached with scales that have fewer intervals (c.f. Friedman and Amoo, 1999).

## Development of our Decision Tree Based Originality Scoring Metric

### Refining the Originality Metric

Despite the potential risk of insufficient correlations between raters, Srivathsavai et al. (2010) argued that the simple agreement levels of their 3- and 4-point scales, as well as the ability to use the modified CEDA metric to evaluate the originality of a creative product in relation to existing products in the marketplace, justified the use of the scale in further research. In further research from the same group, Genco et al. (2011) used a 5-point version of the modified CEDA originality metric to rate the creativity of alarm clock concepts. Genco et al. (2011) reported a kappa of 0.67 between two raters for 10 concepts, which Landis and Koch (1977) called a substantial level of agreement. Kappa is also considered to be a stricter method of inter-rater agreement than correlations or simple agreement (Cohen, 1968; Gwet, 2014), thus showing the improvement of the 5-point scale over the 3- and 4-point scales used by Srivathsavai et al. (2010). Likewise, this modified 5-point originality metric was used by Johnson et al. (2014) with alarm clocks and with litter collection systems. Johnson et al. (2014) reported kappas of 0.90 and 0.70 for the alarm clock and litter collection system concepts, respectively, with two raters independently scoring approximately 45 of each creative product type. Our group also used the modified originality metric with alarm clocks (Kershaw et al., 2014). We reported a kappa of 0.70 between two raters for 20 concepts. This collection of findings (Genco et al., 2011; Johnson et al., 2014; Kershaw et al., 2014) shows that the 5-point modified CEDA originality metric was successfully used to evaluate creative products for two different design problems produced by students at different levels of the curriculum and from different institutions.

The data reported in this article focus on student-generated concepts for next-generation litter collection systems. While we had success in using the modified 5-point CEDA originality metric to evaluate alarm clock concepts (Kershaw et al., 2014), we had difficulty in applying it to the litter collection systems. Kappas between the first and third authors, and between two research assistants, remained low (κ = 0.09–0.42) despite several rounds of training and discussion. There were several potential reasons for these low levels of agreement. One reason was differences we discovered in the instructions that were given to participants: students at one university were told that the litter collection systems were to be used by volunteers doing highway beautification projects (cf. Johnson et al., 2014), while students at another university were not provided with target users for their concepts. Not being provided with target users led to a wider variety of generated products, some of which did not align well with a previously created list of litter collector features. Another reason for the low levels of agreement could be due to this list of litter collector features and its use in evaluation of the concepts. Prior research showed that evaluating originality based on features rather than the overall concept led to better agreement (Srivathsavai et al., 2010), and thus the feature-based evaluation procedure was followed by Johnson et al. (2014) and Kershaw et al. (2014). While multiple feature categories were generated for the litter collection systems, such as how the device harvests litter (garbage interface), its mobility, how a user triggers garbage collection, its storage components, and the overall architecture of the system, most of the variability in originality scores only came from two features: garbage interface and actuation. We began to question if it was necessary to decompose a product into features and evaluate the originality of each feature, or if we could evaluate creativity more globally.

### Development of the Decision Tree for Originality Assessment in Design (DTOAD)

The first two authors, along with two research assistants, made a further modification to the 5-point originality metric by developing a decision tree to aid in the originality evaluation process. Decision trees are a common tool in business, medicine, and machine learning (Goodwin and Wright, 2004) to assist in problem solving. Decision trees are effective for the reasons that diagrams in general are often effective (cf. Larkin and Simon, 1987) – they simplify cognitive operations by providing an external representation of a problem space. Cheng et al. (2001) concur with the cognitive offloading that is afforded by an external representation, and suggest that the most effective diagrams limit the size and complexity of the search that would be necessary to solve a problem or make a decision.

**FIGURE 1:**
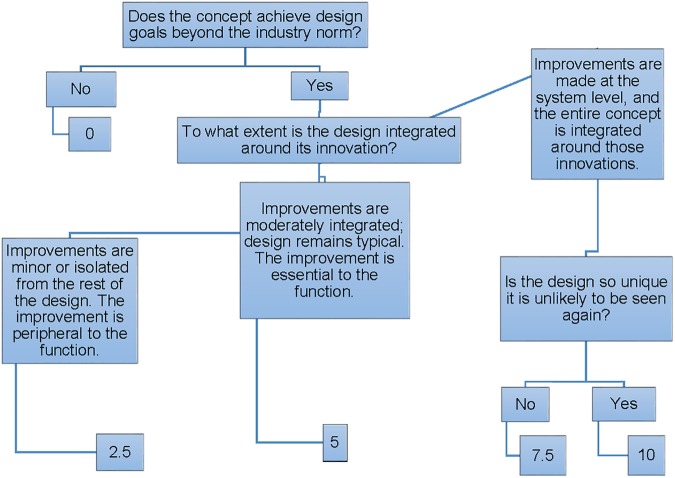
The decision tree for originality assessment in design (DTOAD). © Kershaw et al., 2015, reproduced with permission.

In developing our decision tree for originality assessment in design (DTOAD), we went through several iterations. One of the first versions of the metric focused on how concepts alleviated design flaws. We also originally developed different versions of the decision tree for different types of designs, such as personal litter collectors vs. industrial systems. As noted by Goodwin and Wright (2004), decision tree development is often iterative, just like the development of other types of coding schemes (cf. Chi, 1997). In the end, our final version of the DTOAD incorporated principles from other creative product evaluation methods. First, we kept the 5-point originality metric developed by Genco et al. (2011) based on its past success in describing student-generated concepts. Second, we applied this metric to evaluating the overall concept, rather than its features, to be more in line with the approach taken by the CAT (Amabile, 1982) and the CEDA (Charyton, 2014), as well as other creative product evaluation metrics like Cropley and Kaufman’s (2012) revised Creative Solution Diagnosis Scale. The DTOAD differs from these previous approaches by (a) using a diagram to assist with the originality evaluation process and (b) focusing on how integral design innovations are to the overall concept, rather than parsing a concept into features. The final DTOAD is shown in Figure 1. A description of how we train coders to use this protocol and examples of scored concepts at each level of the decision tree follow in the next section.

## Applying the DTOAD: Full Protocol

### Training Coders to Use the DTOAD

In applying the DTOAD to the scoring of creative products, we follow several guidelines from the literature about how to train coders. First, anyone evaluating the originality of the creative products must become familiar with the coding scheme and the domain from which the products are drawn. As noted by Chi (1997), having an established scheme that is understood by the coders is necessary before coding begins. To establish this familiarity, the coders review the decision tree (see Figure 1) and common features of available products that solve the specified design problem (e.g., the most common features of consumer alarm clocks) and then work together to apply it to a small set of concepts (approximately 10 or so) that have already been scored for originality. The obtained originality scores are then compared to the scores that were already established, and any discrepancies are discussed, thus following a procedure established by Storme et al. (2014) to provide feedback to coders.

Second, the coders independently rate a set of previously coded concepts for originality, blind to curriculum level or any other conditions. The coders’ scores are compared to each other and to established scores. By again providing coders with a comparison to established scores, we help them to develop a schema of how to judge the creativity of the target creative products (cf. Dollinger and Shafran, 2005; Storme et al., 2014). If the coders have reached a sufficient level of inter-rater reliability with the established codes, they are ready to move onto the next step. If not, this process is repeated until a sufficient level of inter-rater reliability is achieved (cf. Chi, 1997). It usually takes coders 2–3 rounds to reach a sufficient level of inter-rater reliability (e.g., Kershaw et al., 2015; Simmons et al., 2018).

At this point, the reader may be asking what a sufficient level of inter-rater reliability is, and how large a sample size must be to reach a sufficient level. Several researchers (e.g., Landis and Koch, 1977; Fleiss, 1981) have published benchmarks for appropriate levels of kappa. Fleiss (1981) called a kappa of above 0.75 “excellent,” and Landis and Koch (1977) noted that a kappa between 0.61 and 0.80 was “substantial.” Neither Fleiss nor Landis and Koch, however, provide guidelines for the sample size needed to establish a reliable level of kappa. Cantor (1996) suggested a well-known set of guidelines for the necessary sample size, but unfortunately his guidelines (as well as those of Gwet, 2014), are based on having only two coding categories (such as deciding that a product is creative or not). As noted above, we are using a 5-point scale. Thus, to determine a sufficient level of inter-rater reliability, we rely on two guidelines: we make sure to reach a kappa of at least 0.7 to meet Fleiss’ (1981) benchmark and we make sure that this kappa is reached through scoring at least 20% of the sample, a common practice in cognitive psychology (Goldman and Murray, 1992; Nye et al., 1997; Chi et al., 2008, 2018; Braasch et al., 2013; Muldner et al., 2014; Kershaw et al., 2018). In our newest work, we also make sure to report the standard error and the 95% confidence interval so that the precision of our kappa values are known (cf. Gwet, 2014).

Once a sufficient level of inter-rater reliability is achieved between the coders and the established scores, we move to the third step of our training procedure. The coders each independently code a subset of the target creative products, i.e., those products that do not already have established originality ratings. As in the second step, coders are blind to condition when rating the creative products. Like in the second step, we again compare the coders’ ratings to see if a sufficient level of inter-rater reliability has been reached. If we have a kappa of at least 0.7, with a low standard error and a confidence interval that only contains acceptable kappa levels, and this level is achieved for at least 20% of the target creative products, then we know that one coder can proceed to code the rest of the set. This coder remains blind to condition as s/he rates the concepts. If we do not have a sufficient level of kappa or have not coded enough creative products, then this process is repeated until we have established inter-rater reliability (cf. Chi, 1997).

### Examples of Coded Data at Each Level of Originality

**FIGURE 2:**
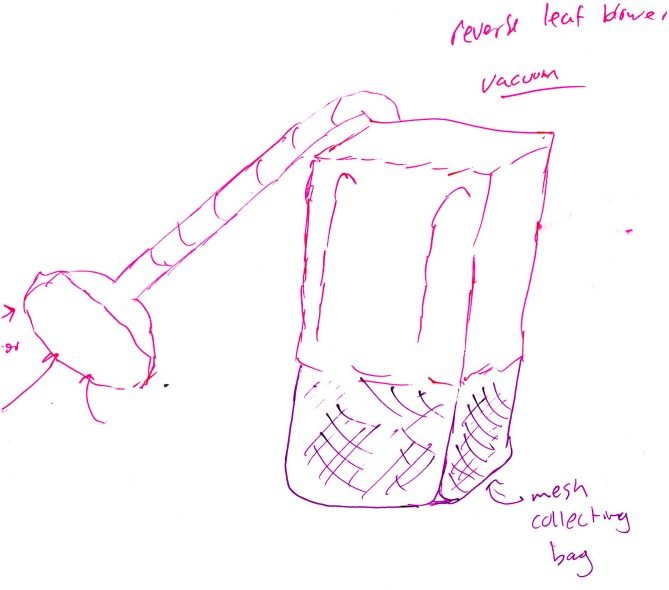
A backpack vacuum that received an originality score of 0.

The DTOAD is shown in Figure 1. First, a coder must decide if the concept achieves design goals that are beyond the industry norm. That is, does the creative product embody any features or solutions that are different from current market products? Recall that the coders were originally exposed to the basic litter collection products available in the market. If it does not, then the product receives a 0 for originality and the coder stops. This category included two main types of designs: designs that were almost identical to the example provided (for cases where the example was provided) and designs that resembled a product used in the market. For example, Figure 2 shows an example of a backpack vacuum system. Based on Figure 2, it is clear that the student essentially chose a leaf-blower system with a vacuum pump replacing a blower. However, this is an existing product, and thus this concept does not differ significantly from current market products. The student is essentially reproducing prior knowledge.

**FIGURE 3:**
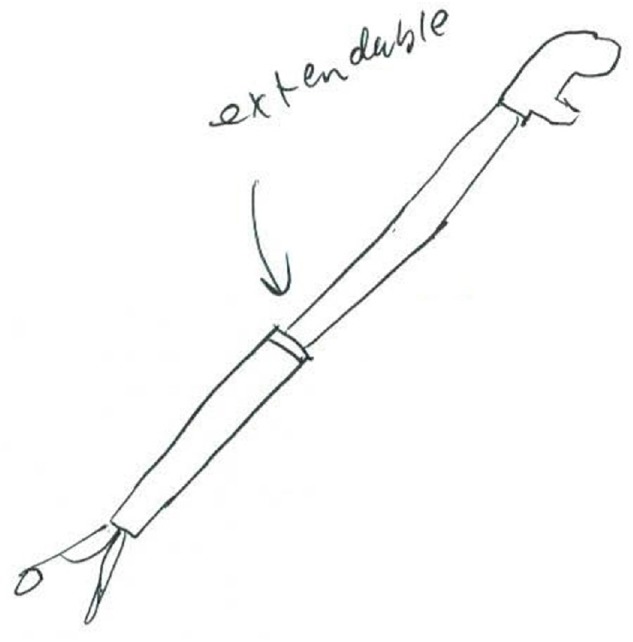
An extendable litter picker that received an originality score of 2.5.

If the creative product embodies features or solutions that extend beyond current products, then the coder must decide the extent to which the concept is integrated around those innovations. If the nature of the new feature is minor, isolated from the rest of the design, or peripheral to the function of the product, then it would receive a 2.5. For example, Figure 3 shows a personal litter picker that can extend. The litter picker is identical to the design of a standard picker, except the flexibility to extend or contract the length of the shaft to desired length. This telescoping modification allows for a longer reach when using the product, but otherwise the concept is equivalent to market products. This is not a fundamental design alteration that would be a new mode of litter collection.

**FIGURE 4:**
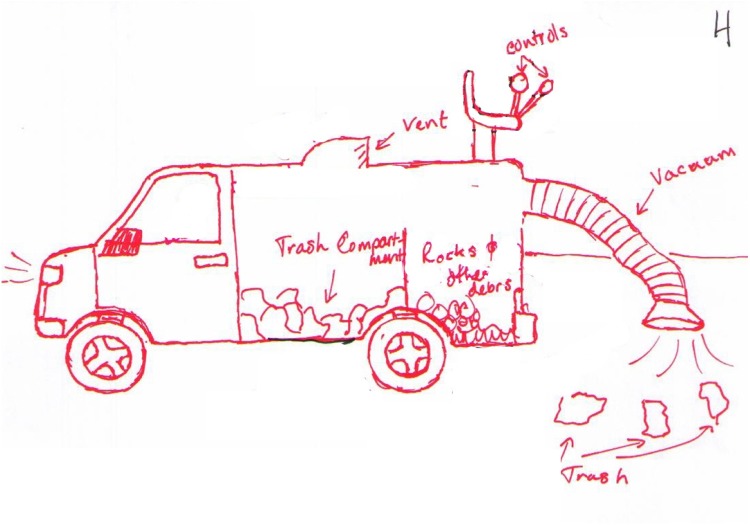
A modified trash truck that received an originality score of 5.

If the new feature entails a moderate level of integration and is essential to the function of the product, yet much of the product’s design remains typical, it would receive a 5. For example, Figure 4 shows several new features that have been incorporated into a garbage truck: it has a vacuum hose that extends from the back, and a means to sort rocks and debris from the trash inside of the truck. The overall architecture of the design is a garbage truck, which is a typical design for a large mobile garbage collection system, however, the atypical placement of the vacuum hose and the internal filtering system show moderate integration with the overall product and are essential to its function, which therefore enhance the overall design.

**FIGURE 5:**
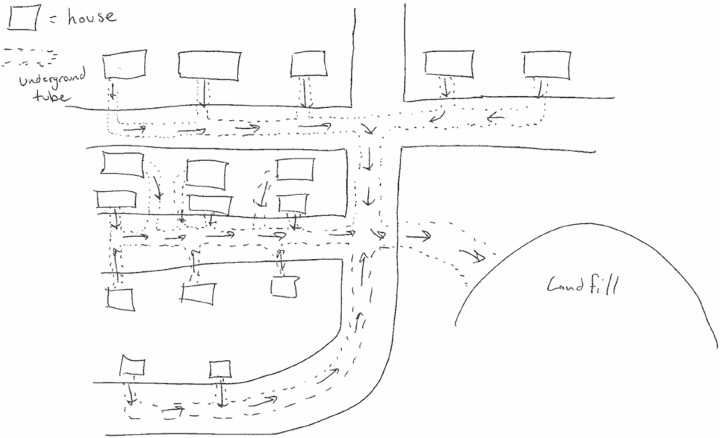
An underground, community-implemented litter collection system that received an originality score of 7.5.

**FIGURE 6:**
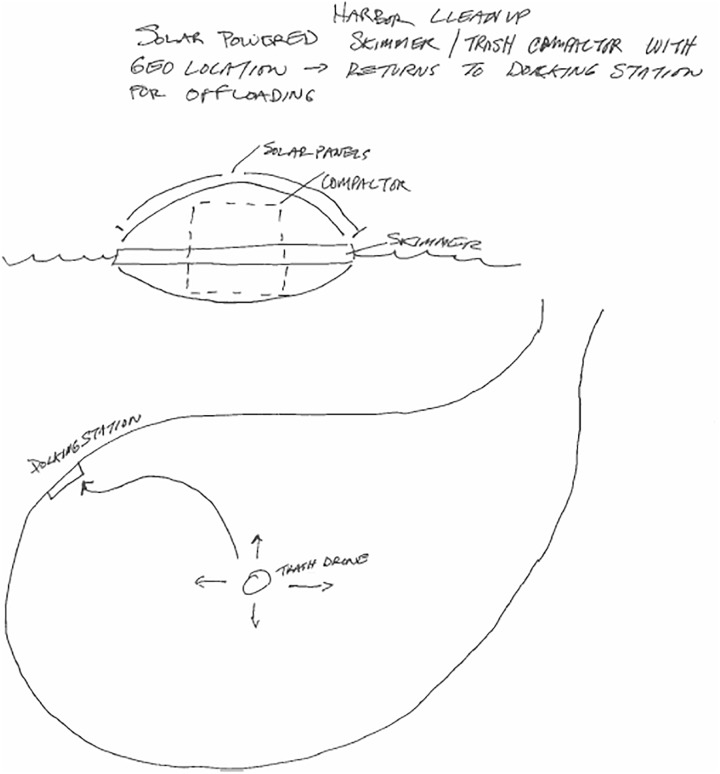
A solar-powered trash skimmer that cleans up harbors that received an originality score of 10.

When the new features are at a system-level, and the entire concept is integrated around those innovations, then the creative product can receive a 7.5 or a 10, depending on the likelihood of seeing the product again. For example, Figure 5 shows a trash collection system that could be used in a neighborhood. Underground tubes carry trash from each home on a street directly to a landfill. This concept displays a unique way of collecting garbage that could be integrated into other infrastructure within a town, such as existing underground water or sewer systems. While this concept shows unique system-level innovations relative to typical litter collection systems, it has appeared several times within our data sets. In contrast, Figure 6 shows a unique device that collects litter from bodies of water, such as a harbor. This floating drone skims trash from the water and compacts it, and then returns to a docking station to deposit the trash and recharge. This concept requires multiple system-level innovations that are not present in current litter collection systems. While autonomous robotic vacuum cleaners are available on the market, they are generally for in-home use and do not contain a compactor. The device in Figure 6 is designed specifically for water use, filters trash rather than vacuuming it, and uses geolocation to return to its “home.” We have not seen a comparable design concept among all the concepts we have coded so far.

## Validating and Using the DTOAD

As described above, and shown through Figures 2–6, the DTOAD has primarily been used to evaluate the creativity of litter collection system concepts. We have also used Shah et al.’s (2003) technical feasibility metric to rate each concept. Technical feasibility has been generally high across concepts (e.g., 9.67 out of 10 for 569 concepts; Kershaw et al., 2015) and we have not found any differences in technical feasibility based on curriculum level (Kershaw et al., 2015) or experimental manipulation (Johnson et al., 2014). Thus, our focus in this paper is on the originality of produced concepts. In applying the DTOAD, we have evaluated undergraduate students across all levels of the mechanical engineering curriculum at the University of Massachusetts Dartmouth and have compared their originality at an overall concept level and at the level of individual contributions to concepts, as well as making cross-sectional comparisons across the curriculum and tracking longitudinal changes in creativity (Kershaw et al., 2015). Much of the previous research summarized in this section was collected using the modified 6-3-5 procedure (Otto and Wood, 2001), in which students are placed in non-interacting groups of approximately 6 individuals. Each student interacts with a sample product (e.g., a personal litter picker) and is asked to generate three ideas. These ideas then circulate through the group so that each student can comment on and modify the ideas of other group members. The ideas circulate through the group five times, or until they come back to the concept originator. While our preliminary work was done following the 6-3-5 procedure, some of our more recent work involved individuals designing on their own with no inputs from others after the preliminary design (cf. LeGendre et al., 2017). The reason for this change in procedure is that Kershaw et al. (2016) found that the concept originator contributed most to the overall originality of a concept. Further, Simmons et al. (2018) showed that there was no difference in the originality level of concepts produced via the 6-3-5 method and those produced using individual ideation. In the following sections, we summarize the results of our previous work (Kershaw et al., 2015, Kershaw et al., 2016; LeGendre et al., 2017; Simmons et al., 2018), then re-analyze a number of litter collection system concepts to reflect what we have learned. We then apply the DTOAD to a different design problem.

### Summary of Previously Published Results

A big part of developing the DTOAD as a firm basis for engineering design creativity coding was to establish inter-rater reliability between coders. As described in Section “Training Coders to Use the DTOAD,” the protocol followed by Kershaw et al. (2015) involved coding of concepts using the DTOAD by multiple coders (three in this case), followed by discussion and clarification to reach convergence. As mentioned above, all coders were blind to condition during the coding process. After each round, the reliability between raters was evaluated using Cohen’s (1968) weighted kappa. Once we achieved a kappa above 0.7, the remaining concepts could be coded reliably (cf. Fleiss, 1981). In Kershaw et al. (2015), 90 of the 569 concepts produced by the participants were coded by three raters, yielding a kappa of 0.73. After this training round, the remaining concepts were coded by a research assistant. This process was then repeated at the individual level for each concept. Each individual’s contribution to each concept, both those that s/he originated and those that s/he modified through the 6-3-5 process, was scored for originality using the DTOAD. To establish inter-rater reliability, three raters coded the contributions of 35 individuals to 90 concepts, yielding κ = 0.85. A research assistant then coded the remaining individual contributions.

Our first work using the DTOAD explored engineering creativity across the curriculum (Kershaw et al., 2015). Cross-sectional analysis of results was performed at both the overall and individual level, examining 569 concepts produced by 242 individuals. Our first goal was to ascertain whether we could find any difference in creativity across the curriculum in Mechanical Engineering cross-sectionally. We did not find a significant difference between the 4 years (freshmen, sophomores, juniors, and seniors), either at the concept or individual contribution level. A follow-up analysis comparing extreme groups (freshmen vs. seniors) showed no significant difference between these groups, but some significant differences within the groups, such that seniors tested at the end of the school year had higher originality scores than those tested at the beginning of the school year. There was not a significant change in freshmen’s originality. This pattern in the extreme groups was shown at both the concept and individual contribution level.

In the same paper, another set of analyses assessed longitudinal differences with students who were tested multiple times during the undergraduate curriculum (Kershaw et al., 2015). Specifically, the concept-level and individual-level litter collection system originality scores were compared within a small group (*n* = 7) of students who were juniors during the Fall, 2012 semester and seniors during the 2014 semester. We found that originality significantly increased from the beginning of the junior year to the end of the senior year without any changes in self-reported GPA or self-reported engineering design self-efficacy. In summary, an improvement in design creativity was observed from the junior to the senior year, with seniors showing some of the highest originality. Although we did not see the cross-sectional results shown in other design problems collected at the same institution (cf. Genco et al., 2011; Kershaw et al., 2014), we were able to establish inter-rater reliability, thus providing some confidence in using the DTOAD for evaluation of engineering creativity.

As mentioned above, the concepts in Kershaw et al. (2015) were collected using the modified 6-3-5 method (Otto and Wood, 2001). In Kershaw et al. (2016), we examined the effects of within-group processes on originality. We used 290 freshman and senior concepts from Kershaw et al. (2015) that received originality scores of 2.5 or higher to examine the weight of each group member’s contribution to the originality of a concept. We classified the top scoring contributor of each concept as the originator of the concept, a different group member of the same group, or multiple members of the same group with the same originality score (Kershaw et al., 2016). We found that the majority of concepts produced (73%) had the concept originator as the top contributor. Further, a comparison of originality scores between these three types of top contributors indicated that groups in which the concept originator was the top contributor had higher originality scores than groups in which a different group member was the top contributor. There were no other significant differences between the contributor types.

While the only significant difference in concept originality in Kershaw et al. (2016) was between the concept originator and a different group member as the top contributor, the large percentage of concepts in which the originator was the top contributor pointed to a possible limitation of group design exercises like the 6-3-5 method. Since the majority of the creativity came from design originators, it is possible to argue that the subsequent contributors fixated on the originator’s design and did not contribute anything new. Thus, for our next paper (LeGendre et al., 2017), students generated litter collection system concepts individually, i.e., they completed individual ideation but did not work in groups nor make contributions to other concepts within a group. Further, unlike our previous work, students in LeGendre et al. (2017) did not receive a sample product with which to interact prior to the ideation phase.

Simmons et al. (2018) compared these individually generated concepts from LeGendre et al. (2017) to concepts that were collected using the 6-3-5 method. The first and second author, along with two research assistants, established inter-rater reliability by first reviewing sets of concepts (34 from the group-ideation set and 35 from the individual-ideation set) and then independently coding additional concepts. Thirty (18%) of the group-ideation concepts and 39 (21%) of the individual-ideation concepts were coded by the first and second authors and the research assistants, yielding kappas of 0.79 for the group-ideation concepts and 0.84 for the individual-ideation concepts. The research assistants then coded the remaining concepts. For analysis purposes, only the concept originator scores were used for those concepts collected using the 6-3-5 method. Simmons et al. (2018) found a difference between concepts generated by seniors and freshmen, such that seniors had higher originality scores. They did not, however, find any difference in originality scores between concepts generated through the individual-ideation and group-ideation methods. This result shows us that the DTOAD can be used when concepts are produced by individuals or by groups, and when students are provided with an example product or not prior to ideation. Further, it shows us that similar levels of originality are reached whether an example product is provided or not.

### Re-analysis of Litter Collection System Concepts

Over the course of multiple years, we have collected data from over 450 students who have produced over 1000 l collection system concepts. Our original aim with collecting these concepts was to assess differences in creativity between students at different points in the undergraduate mechanical engineering curriculum. In the following re-analysis, we focus on groups of students who are at opposite ends of their undergraduate careers, and for whom we have the most data: freshmen and seniors. In selecting the concepts for this re-analysis, we chose concepts produced by students in the spring of their respective year. For the freshmen, this would be the first course that focused on their specific sub-field of mechanical engineering. For the seniors, this would be the last course that is the culmination of their undergraduate training: senior design. Because Kershaw et al. (2016) found that the bulk of the originality score of a given concept came from the concept’s originator, we only used concept originator scores for this analysis. Likewise, because Simmons et al. (2018) found no difference in originality between concepts that were produced via the modified 6-3-5 procedure (Otto and Wood, 2001) and concepts that were produced via individual ideation, we include concepts that were produced via both methods.

Based on the above criteria, we selected 420 concepts that were produced by 216 freshmen and 318 concepts that were produced by 141 seniors. These concepts had already been scored for originality and had been part of the analyses in their respective publications (Kershaw et al., 2015, Kershaw et al., 2016; LeGendre et al., 2017; Simmons et al., 2018). An examination of distribution of originality scores led to the removal of two outlying scores, one from the freshman concepts and one from the senior concepts, which were more than three standard deviations above the mean. Thus, 419 freshman concepts and 317 concepts were analyzed. An independent-samples t-test indicated that seniors (*M* = 2.47, *SD* = 2.42) produced concepts that were more original than freshmen (*M* = 1.77, *SD* = 2.04), *t*(734) = -4.27, *p* < 0.001, *d* = 0.31. These results support the findings of several other studies that have shown that advanced students display higher levels of creativity than beginning students (Cross et al., 1994; Ball et al., 1997; Atman et al., 1999; Kershaw et al., 2014).

### Applying the DTOAD to a New Design Problem

Our summary of previous data and re-analysis of the litter collection system concepts show how the DTOAD metric can be applied to concepts that are produced by individuals with different levels of engineering knowledge (freshmen vs. seniors). We have also shown how the DTOAD can be applied at both the concept and individual level (Kershaw et al., 2015) for group-produced concepts. Further, we have shown how the DTOAD can be applied for concepts produced both within a group setting and individually, with and without an example product (Simmons et al., 2018). All of these applications, however, have been with the litter collection system design problem. In this section, we apply the DTOAD to a different design problem, in which students were asked to generate ideas for next-generation thermometers.

The data in this section were collected as part of a master’s thesis (Genco, 2012). Participants, all senior mechanical engineering students, experienced the modified 6-3-5 procedure (Otto and Wood, 2001). All students began by interacting with two sample thermometers, one that measured temperature under the tongue and one that measured temperature by holding it to a person’s forehead. All students were given up to 30 min to interact with the thermometers to understand their function. Students in an experimental group interacted with the thermometers while using devices that were meant to mimic sensory impairments, such as limited vision, hearing, and dexterity. To mimic limited vision, participants wore blindfolds while interacting with the thermometers. To mimic limited hearing, they wore headphones while interacting with the thermometers, and to mimic limited dexterity, they wore oven mitts. Students in a control group simply interacted with the thermometers without using the disabling devices. The experimental conditions were designed to engage the participants in empathic experience design, a structured conceptual design method focusing on stimulating user-centered concept generation by engaging designers in empathic experiences. Empathic experiences are demanding product interaction tasks that simulate actual or situational disabilities experienced by lead users of a product. The goal is to help the designer empathize with these lead users and design products that better meet their needs and requirements. This study focused on evaluating the effectiveness of empathic experience design. Genco et al. (2011) had previously shown that empathic experience design increased the novelty of alarm clock concepts, and Johnson et al. (2014) showed a similar finding for litter collection system concepts.

The first and second authors, along with a research assistant, used the DTOAD metric to code the thermometer concepts, blind to condition. Due to the small number of concepts (*n* = 41), we did not follow our usual procedure of establishing inter-rater reliability and then having one coder complete the originality scoring. Instead, each coder scored all the concepts. Disagreements were resolved and the team decided on a final originality score for each concept.

To make the analysis of the thermometer concepts similar to that of the re-analyzed litter collection system concepts, we used the concept originator’s scores in the following analysis. Of the 41 concepts that were coded for originality, only two included group contributions that would have been scored as original beyond the concept originator’s idea. An initial examination of the distribution of originality scores indicated one score within the control group that was more than three standard deviations above the mean originality score for this group. After the outlying score was removed, an independent samples *t*-test was conducted, *t*(38) = -2.06, *p* < 0.05, *d* = 0.66. Concepts produced in the empathic experience design groups had higher originality scores (*M* = 2.26, *SD* = 1.92, *n* = 21) than concepts produced in the control groups (*M* = 1.18, *SD* = 1.28, *n* = 19). The results for the thermometer concepts using the DTOAD replicate other creativity results using Shah et al.’s (2003) metric to analyze concepts produced through the empathic experience design procedure (Genco et al., 2011).

## Advantages and Limitations of the DTOAD

### Advantages

There are several advantages to the DTOAD. First, it is a reliable instrument for the measurement of creativity, as shown through the high levels of inter-rater agreement reached between coders (see Summary of Previously Published Results). The training process we follow with our coders (see Examples of Coded Data at Each Level of Originality) allows them to recognize original creative products. Second, the DTOAD shows a high degree of construct validity. It shows convergent validity (cf. Cronbach and Meehl, 1955) with other evaluation instruments of creative products: fixation scores (Jansson and Smith, 1991; Vasconcelos and Crilly, 2016) and Shah et al.’s (2003) novelty metric. The DTOAD shows discriminant validity with other measures of creativity, such as the Remote Associates Test (RAT) (Mednick, 1962; Smith and Blankenship, 1991).

#### Relationship to Fixation

LeGendre et al. (2017) examined the relationship between fixation and originality within the litter collection system concepts that were reported in Kershaw et al. (2015). The fixation metric measured the presence or absence of each repeated feature of the example product (see Table 1), following the procedure of Jansson and Smith (1991). This replicated features measure of fixation is common in the literature: over half of the studies included in Vasconcelos and Crilly’s (2016) meta-analysis used a replicated features measure of fixation. LeGendre et al. (2017) found a significant relationship between fixation and originality, *r*(729) = -0.21, *p* < 0.001. Using a new set of litter collection system concepts, Simmons et al. (2018) found a similar negative relationship between fixation and originality, *r*(243) = -0.32, *p* < 0.001. For example, Figure 3 shows a litter picker that replicates four features of the provided example: a pistol trigger, an unbroken long rod, a prong quantity of two, and a prong end. As noted in Section “Examples of Coded Data at Each Level of Originality,” this concept received an originality score of 2.5, thus illustrating the negative relationship that LeGendre et al. (2017) and Simmons et al. (2018) found between fixation and originality. It is important to note, however, that Simmons et al.’s (2008) results were only shown when participants were provided with an example litter collector to interact with prior to ideation. When no example litter collector was provided, there was no longer a significant relationship between fixation and originality (*r*[154] = -0.01, *p* = 0.44).

**Table 1:** Feature descriptions for fixation coding.

Design feature	Description
Pistol Trigger	Any trigger with a handle and pull mechanism resembling a square and a line.
Unbroken Long Rod	A fixed length that cannot be changed which connects the trigger to actuator.
Prong Quantity	Any two or three component grabbing structure acting as the picker, i.e., claws, cups, or plates.
Prong End	Any shape or line at the end of a prong.
Hand Support	Any small shape that is connected to the hand grip in order to add ergonomic support.
Locking Mechanism	Any indication of a locking mechanism, i.e., text or shape similar to the lock on the example.


The negative relationship between fixation and originality found by LeGendre et al. (2017) and Simmons et al. (2018) is expected given the nature of these measures. Creative products that are deemed original should not show a high degree of design fixation. These fixation results show convergent validity between the DTOAD and common ways (cf. Vasconcelos and Crilly, 2016) of measuring fixation.

#### Relationship to Novelty

**FIGURE 7:**
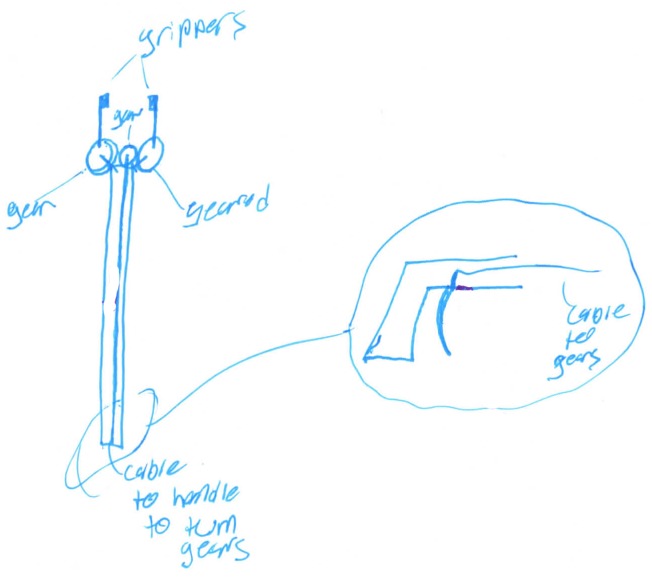
A litter picker that received low originality and low novelty scores.

Additional support for the construct validity of our conception of originality is shown through convergent validity with Shah et al.’s (2003) novelty metric. The first and fourth author chose a subset of 185 freshman, junior, and senior concepts from the set coded for originality by Kershaw et al. (2015). Following Shah et al.’s (2003) guidelines and the procedures used by Srivathsavai et al. (2010), we first decomposed the litter collection systems into features based on functions including the means of collecting trash (garbage interface), mobility of the system, and its actuation (trigger to collect garbage; see Table 2 for all features). Next, we developed labels of the expression of each feature based upon what was present in the dataset. For example, the possibilities for trash treatment within our sample included that garbage was stored within the system, that there was separate storage, or that garbage was burned, compacted, recycled, or ground. We also included a label of “none” for when a means of trash treatment was not included within a concept, and a label of “not clear” for when it was impossible to determine the means of trash treatment for a given concept (see Table 2 for all expressions within each feature). After developing a final set of features and expressions within those features, the first and fourth author coded the chosen concepts by describing the expression of each feature within each concept. For example, Figure 7 shows a litter collection system that is a litter picker (architecture) with a claw that collects trash (garbage interface) when a human (control) squeezes its handles (actuation) via manual power. This litter collector is carried (mobility) by a person but no modifications were made to this design in consideration of its intended user. This concept does not include any means for trash treatment or removal within it.

**Table 2:** Litter collection system features and their expressions for novelty coding.

Feature	Garbage interface	Mobility	Actuation	Trash treatment	Trash removal	Power source	Control	User considerations	Architecture
Expressions	Vacuum	Carried	Button	Stored (in device itself)	Removable bag	Manual	Human	Personal item storage	Standard design
	Suction	Worn	Switch	Separate storage	Door access	Human, power stored	Automated	Safety cabin	Novelty shape
	Shovel	Rolling robot	Lever(s)	Burned	Zipper to access	Hydropower	None	Body strap	Extends
	Claw	Vehicle	Squeeze handle(s)	Compacted	Vibrate to shake trash off picker	Pneumatic	Not clear	Easier handles	Body extension
	Reverse claw	Pulled by vehicle	Trigger	Recycled	Release button	Battery (cordless)		Light weight	Folds for storage
	Brushes rolling	Push cart	Continuous	Grind	Push trash off collector	Electric (corded)		One-handed squeeze	Multiple effectors
	Water flow	Pushed or pulled sweeper	Manual motion	None	Dump	Gasoline		Mechanical assist	Modular system
	Spear	Fly	Senses trash	Not clear	Not clear	Solar		Padding	Distributed system
	Conveyor belt	Stationary	None		None	Wind		Grip	Integrated system
	Sticky pad	Not clear	Not clear			Fuel cell		Vision	Infrastructure
	Magnet					Biofuel		Weight distribution	
	Laser					Nuclear		Customized	
	Flap					Not clear		None	
	Net								
	Human hand								
	Robot hand								
	Animal								
	Not clear								


After all the selected litter collection system concepts were coded, we compared all described features of a given creative product to the range of features expressed within a set of products to determine its novelty score. Shah et al.’s (2003) novelty metric can be used to measure the uniqueness of a particular feature within a set of creative products, or can provide overall measures of the novelty of a creative product by averaging the uniqueness of all features (average novelty) or choosing the highest novelty score of a feature from each concept (maximum novelty). For the purposes of comparing novelty to originality, we chose the maximum novelty measurement to ensure that creative designs were not stifled by containing standard features, and because the DTOAD considers the integration of innovative features beyond the industry norm. We found a significant positive correlation between originality and maximum novelty, *r*(185) = 0.35, *p* < 0.001. This positive relationship would be expected given that both the DTOAD and Shah et al.’s (2003) novelty metric are designed to assess the creativity of ideas. At the same time, it is important to note that this correlation is moderate – the two metrics are not measuring creativity in the same way.

**FIGURE 8:**
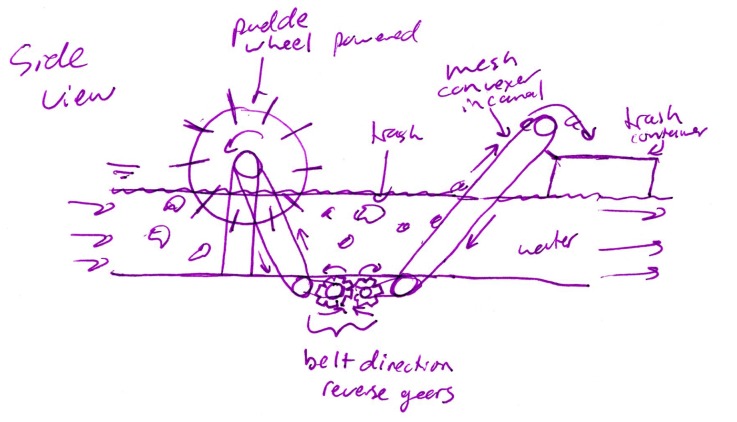
A paddle wheel and conveyor water-based trash collection system that received high originality and high novelty scores.

**FIGURE 9:**
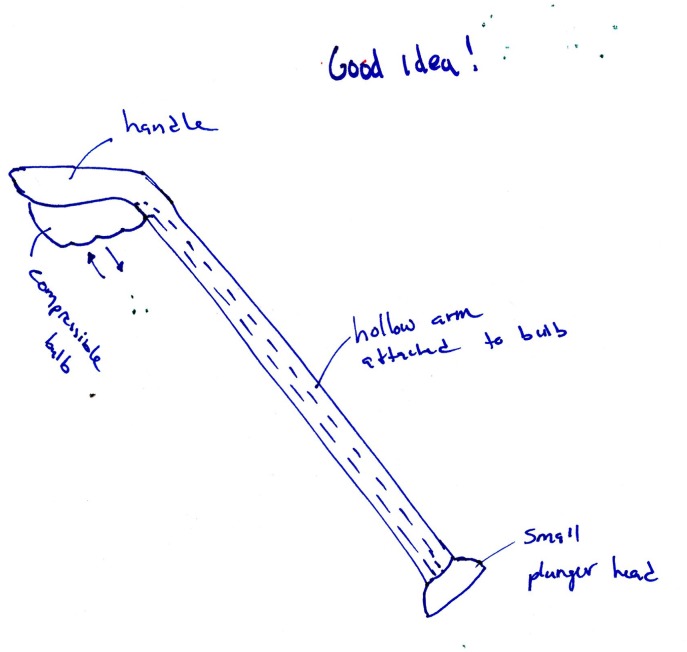
A picker with a suction cup end that received a low originality score but a high novelty score.

Figure 7 shows an instance when the originality and novelty metrics agree – this concept has low maximum novelty (6.47 out of 10), and a score of 2.5 on the DTOAD for minor improvements to a function of a typical litter picker. Figure 8 shows another instance of agreement between the metrics. This concept received an originality score of 7.5 using the DTOAD metric because it shows system-level integration of multiple features, including the use of water currents for powering the device and enabling filtration. Within the set of concepts chosen to measure novelty, the water wheel filtration system shown in Figure 8 contained five rare features, including the use of hydropower, being a stationary system, and its overall atypical architecture, thus boosting its novelty score to 9.88. In contrast, Figure 9 shows an instance of disagreement between the two metrics. Any disagreements we found between the metrics occurred when the novelty metric indicated that a concept was unique and the DTOAD did not. The reverse circumstance did not occur. For example, the concept shown in Figure 9 received a 2.5 using the DTOAD because it only displays a small modification of using suction instead of a claw to collect trash within a typical litter picker design. In contrast, the concept shown in Figure 9 had a high novelty score (9.88) because the use of suction within the garbage interface feature was rare. As noted in Section “Evaluating Creative Products in Engineering,” Shah et al.’s (2003) novelty metric relies on novelty within a set of creative products (Sarkar and Chakrabarti, 2011) and does not compare creative products within a set to other sets or to current market products (Srivathsavai et al., 2010). Thus, the DTOAD may provide a truer evaluation of the creativity of a design by comparing it to a large set of related designs that are not present within a given set of ideas.

#### Relationship to Remote Association

The DTOAD shows convergent validity with fixation and novelty. These are common ways to assess creativity, but another way to conceive of creativity is through the lens of convergent and divergent thinking (cf. Guilford, 1956; Cropley, 2006). Convergent thinking can be defined as using conventional and logical search strategies to arrive at solutions. While an individual may consider many options, a single solution is usually chosen. In contrast, divergent thinking can be defined as using unconventional and flexible thinking to arrive at solutions. Divergent thinking frequently leads to the production of multiple solutions, or multiple perspectives on a situation or problem. One aspect of divergent thinking is how well individuals can make connections between disparate ideas. It is hypothesized that individuals who are more creative have less steep association hierarchies – that is, concepts in long-term memory are less strongly related than for individuals who are less creative (cf. Mednick, 1962). Having weaker association hierarchies increases the likelihood that individuals will generate novel responses. This aspect of divergent thinking could be useful in creative design because it would allow a person to be more flexible when generating ideas for a creative product. Nijstad et al. (2010) argued that the originality of ideas is highly related to the level of flexibility a person shows by exploring many options during the ideation process. It is important to note, however, that both convergent and divergent thinking contribute to the production of original ideas. Variability alone is necessary, but not sufficient for creativity – convergent thinking is needed to evaluate generated ideas (cf. Cropley, 2006). Well-known models of creativity account for both convergent and divergent thinking, such as Campbell’s (1960) blind variation and selective retention model of creativity (see also Simonton, 2011) or the creative problem-solving framework that is used in educational settings (Treffinger et al., 2006).

To understand the relationship between originality and remoteness of association, we used the RAT, a traditional psychometric creativity instrument. The RAT utilizes both divergent thinking (to explore connections between concepts) and convergent thinking (to choose the most appropriate connection, or answer to the RAT problem). Individuals commonly generate multiple possible connections between the words in a given RAT problem (divergent thinking) before choosing the best solution to the problem (convergent thinking; Wieth and Francis, 2018). Twelve RAT items were chosen from Mednick (1962) and Smith and Blankenship (1991). The RAT asks participants to generate a fourth word that forms a phrase with each of three provided words. For example, if the provided words were *blue, cake*, and *cottage*, a correct generated answer would be *cheese* (*blue cheese, cheesecake, cottage cheese*). The RAT was administered to a subset of senior mechanical engineering students who generated the concepts that were part of Kershaw et al. (2015). We correlated RAT scores with the average originality across the litter collection concepts these students produced. There was not a significant relationship, *r*(23) = -0.13, *p* = 0.55. This result within the litter collection system concepts replicated our earlier findings that RAT scores did not predict originality for alarm clock concepts (Kershaw et al., 2014). This result also supports the findings of Kudrowitz et al. (2016), who found no relationship between the RAT and performance on creative design tasks. Remoteness of association and ideation in engineering design involve both convergent and divergent thinking ability (cf. Jaarsveld and Lachmann, 2017), but the RAT appears to be evaluating a different aspect of creativity than the ability to generate original ideas via creative products, as measured through the DTOAD (cf. Cropley et al., 2017, for a similar discussion).

### Limitations

There are several limitations of the DTOAD as presented in this paper. First, the DTOAD is most appropriate for design problems or applications for which closely related products or solutions are available in the marketplace. These existing solutions serve as benchmarks or anchors for determining whether the newly proposed solutions are different in some way from those benchmark products. On the positive side, it is difficult to identify a design problem for which no benchmark solutions exist. Even products considered revolutionary upon introduction to the marketplace, such as the first smartphones, replaced or augmented existing products performing similar functions, e.g., larger laptop computers. The difficulty is that a thorough and relevant set of benchmark products must be collected prior to application of the decision tree because an incomplete set of benchmark solutions may lead to artificially high ratings for solutions that already exist in the marketplace. Moreover, with a rapidly changing technology landscape, a definition of dynamic creativity that accounts for “potential” originality rather than a fixed scale should be accounted for, as described by Corrazza (2016). The challenge remains to design a coding scheme that accommodates creative inconclusiveness in the context of the existing literature and provides insights for future scientific questions in the field of creativity.

Second, the DTOAD requires a lengthy training procedure. New raters are required to evaluate subsets of concepts and compare their results with expert ratings, and to repeat the procedure until sufficient inter-rater reliability is achieved. Our experience using the DTOAD, and training our research assistants, was that it was easier to identify highly creative products (typically assigned a 7.5 or 10 in our decision tree), but challenges were presented in the lower end of the scale (0–5). Understanding whether a product is radically different and therefore creative (e.g., receiving an originality score of 7.5 or 10) is not difficult. This is possibly why the CAT (Amabile, 1982) has been such a successful tool in non-engineering fields (cf. Baer and McKool, 2009; Beaty et al., 2013; Kaufman et al., 2013, Study 1). If one analyzes creativity in literature or art, a novice is usually able to identify a high degree of creativity without understanding all the details of the process. Similarly, a coder with no prior engineering knowledge would be able to identify highly creative engineering design for a common product like alarm clock or litter collection system (as opposed to a guided missile system). However, the disagreement in originality scoring that occurred during the training process usually was at the lower end of the spectrum. Although there was broad agreement in scores of 0 where no novel feature was identified, we experienced challenges in separating designs in the 2.5–5 range. While this may not be an issue in business and industry, where the goal is to identify break-through levels of novelty, in research settings it is important that we can distinguish between all levels of originality. There was some disagreement about what constituted a ‘novel’ feature deserving a positive score. Coders also sometimes disagreed on what constituted an ‘isolated’ feature vs. a ‘moderately integrated’ feature. While some engineering knowledge may be helpful (cf. Kaufman et al., 2013, Study 2), perhaps clearer instructions are required to understand integration of features at the system level. To address these issues, we have started tabulating a database for novel features to help future coders.

Similar challenges regarding training coders apply to other creativity metrics, including the 5-point scale utilized in our previous research (cf. Genco et al., 2011; Johnson et al., 2014). Recent work in crowdsourcing, however, suggests that extensive training may not be required. For example, Green et al. (2014) spent 20 min training a large group of novice raters to evaluate concepts for the alarm clock problem discussed previously. Even with such a short training session, they found that novice raters with high inter-rater reliability amongst themselves served as a very good proxy for an expert rater. Large numbers of raters (on the order of 40) are needed, however, to identify raters with excellent inter-rater reliability amongst themselves. Perhaps these raters could be recruited via Mechanical Turk or other similar mechanisms, and the training could be conducted online. The success of this type of crowdsourcing effort may also depend on the raters’ familiarity with the design problem and the raters’ incentives for rating the concepts carefully and thoughtfully.

## Conclusion

The DTOAD metric was an evolution from existing techniques reported in the literature, such as the CAT (Amabile, 1982), Shah et al.’s (2003) novelty metric, and the CEDA (Charyton et al., 2008; Charyton, 2014). The DTOAD also derives from previous modifications to the CEDA by Srivathsavai et al. (2010). It evolved as a part of an interdisciplinary study of engineering creativity conducted by several faculty and students at various universities. A variation of the CEDA (Charyton et al., 2008; Charyton, 2014) was previously used for analyzing alarm clocks (Genco et al., 2011; Johnson et al., 2014). However, we faced considerable challenges with low inter-rater reliability as we tried to use the method for a more complex engineering product like a litter collection system. While not every litter collection system is complex, this design problem requires individuals to generate ideas for creative products for which they have less familiarity as consumers. Other than trash cans and litter pickers, most litter collection systems are not meant for an individual consumer. Due to these challenges, we developed the DTOAD using a five point scale. The specific decision tree method described here for analyzing creativity was developed for analyzing concepts for “next generation litter collection systems” generated by undergraduate engineering students.

The evolution of the DTOAD is an attempt to measure creativity in complex engineering designs that go beyond simple “features” or “variety” or “novelty.” It is an attempt to develop an algorithm for analyzing creativity in complex engineering designs for the future. The former creativity evaluations are useful indicators of creativity, but were not always geared toward evaluation of features as well as the system level integration. An important challenge that exists in analyzing ‘complex’ system level designs for creativity is to have a knowledge of how features are integrated at the system level. It also requires a working knowledge of the product to assess what the industry standards are, not just at the feature level, but at the system level as well. Therefore, considerable effort was expended during the training process of the coders to establish an understanding of the state of the art of the product, its features, and their integration. While analyzing the litter collection system, we did evaluate ‘features’ that were considered novel. However, to get a score of 5, a designer had to demonstrate integration of the ‘feature’ within the existing architecture. Higher scores were typically assigned to novel architectures that went beyond the existing industrial norms.

Despite some of the challenges encountered during the development and implementation of the DTOAD, we have been able to obtain meaningful and insightful trends. We have applied the decision tree to concepts generated by all 4 years of engineering students (freshmen through seniors). When we used the modified 6-3-5 method (Otto and Wood, 2001), we were able to analyze originality at the overall concept level and also at the individual level (Kershaw et al., 2015), as well as examine effects of group processes on creativity (Kershaw et al., 2016). We also have analyzed data for students who were provided an example product as well as students who were provided no examples. Further, we have also applied the DTOAD to evaluating thermometers, a product that was not considered when developing our originality metric. Overall, we have found that the senior mechanical engineering students have a higher originality score than freshmen, reinforcing studies by others reported in the literature (Cross et al., 1994; Ball et al., 1997; Atman et al., 1999). This provides some order of validity to our method of analysis. We have also found that the originator of a design typically has the highest contribution to creativity. We have also found an inverse correlation between originality and fixation (LeGendre et al., 2017; Simmons et al., 2018). Removing the example product lowered fixation drastically, but did not increase originality. In this way we have gained measurable insights into the design creativity thinking process of mechanical engineering students. These results will provide useful data points for curricular design where further creative thinking is required as a part of engineering education.

The mechanical engineering undergraduate curriculum is diverse with significant emphasis on quantitative techniques and set-piece problem solving (Accreditation Board for Engineering and Technology, 2014). Synthesis of concepts from various courses into a holistic design process is limited. Without the experience of synthesis, students are not encouraged to think creatively or perform creative design tasks. One of the goals of creativity research in engineering is to understand how to improve the creative thinking process in the engineering curriculum (Dym et al., 2005; Phase, 2005; Duderstadt, 2010). Although a statistically significant difference was found in the originality scores between freshmen and seniors when measured longitudinally (Kershaw et al., 2015), the overall differences and trends were not drastic to indicate that students were being trained well in the creative process. It is our goal to use the data generated from the studies using the decision tree to propose active measures within the curriculum.

Measuring creativity in engineering design is an extremely important tool beyond academia as well. Establishing a creative toolbox and analyzing creativity are important for companies to develop new products for the future to stay competitive and maintain their cutting edge in an increasingly shrinking market space. In addition to a marketing survey it is becoming imperative for companies to test the “coolness” factor of many consumer products. This evaluation is often related to creative design. However, there are no standard tools available to companies that can measure creativity in engineering design. Therefore, developing creativity measuring tools for engineering design continues to be an important goal in design research (cf. Cropley and Kaufman, 2012).

## Data Availability Statement

The raw data supporting the conclusions of this manuscript will be made available by the authors, without undue reservation, to any qualified researcher.

## Ethics Statement

All data analyzed in this paper were retrieved from previously published or unpublished studies. Those studies were carried out in accordance with the recommendations of the Institutional Review Board (IRB) at the University of Massachusetts Dartmouth. Their respective research protocols were approved by the UMass Dartmouth IRB. All participants were provided with informed consent letters because an exemption from signed consent forms was granted by the UMass Dartmouth IRB. Participants read the consent letter, asked any questions that they had, and then consented to be part of the study by taking part in an in-class activity. Participants kept copies of the consent letter so that they could contact the researchers for additional information at a later date.

## Author Contributions

TK and SB developed the DTOAD based on, in part, previous originality metrics developed by KH-O and CS. TK, SB, and a research assistant completed the originality coding for the thermometer concepts. TK and KH-O completed the novelty coding. TK completed all statistical analyses. KH-O and SB assisted with the interpretation of the results of these analyses. TK wrote the initial draft of Sections “Introduction,” “Development of our Decision Tree Based Originality Scoring Method,” “Applying the DTOAD: Full Protocol,” “Re-analysis of Litter Collection System Concepts,” “Applying the DTOAD to a New Design Problem,” and “Advantages.” SB wrote the initial drafts of Sections “Summary of Previously Published Results” and “Conclusions.” CS wrote the initial draft of Section “Limitations.” KH-O provided helpful comments on all sections. All authors edited all sections of the manuscript, responded to reviewer comments, and approved the final version.

## Conflict of Interest Statement

The authors declare that the research was conducted in the absence of any commercial or financial relationships that could be construed as a potential conflict of interest.
